# Rapid Test Ag 2019-nCoV (PROGNOSIS, BIOTECH, Larissa, Greece); Performance Evaluation in Hospital Setting with Real Time RT-PCR

**DOI:** 10.3390/ijerph18179151

**Published:** 2021-08-30

**Authors:** Maria Kyritsi, Alexandros Vontas, Ioanna Voulgaridi, Alexia Matziri, Apostolos Komnos, Dimitris Babalis, Antonios Papadogoulas, Aikaterini Oikonomou, Varvara A. Mouchtouri, Matthaios Speletas, Christos Hadjichristodoulou

**Affiliations:** 1 Laboratory of Hygiene and Epidemiology, Faculty of Medicine, University of Thessaly, 41500 Larissa, Greece; mkiritsi@uth.gr (M.K.); avontas@uth.gr (A.V.); ioanna.voulgaridi@gmail.com (I.V.); alexmatziri@gmail.com (A.M.); mouchtourib@med.uth.gr (V.A.M.); 2 Intensive Care Unit, General Hospital of Larissa, 41221 Larissa, Greece; komnosapo@gmail.com (A.K.); antonis09pap@gmail.com (A.P.); 3 Emergency Department, General Hospital of Larissa, 41221 Larissa, Greece; dbabales@yahoo.com; 4 Internal Medicine, First Department of Internal Medicine, General Hospital of Larissa, 41221 Larissa, Greece; oikonomoukaterina85@gmail.com; 5 Department of Immunology and Histocompatibility, Faculty of Medicine, University of Thessaly, 41500 Larissa, Greece; maspel@med.uth.gr

**Keywords:** SARS-CoV-2, nucleocapsid protein, rapid antigen test, immunochromatography

## Abstract

Introduction: Rapid antigen tests (RATs) are convenient for SARS-CoV-2 detection because they are simpler and faster than nucleic acid amplification tests (NAATs). This study aimed to assess the accuracy of a locally manufactured test; Rapid Test Ag 2019-nCoV (PROGNOSIS, BIOTECH, Larissa, Greece) in a clinical setting and during mass screening. Methods: Nasopharyngeal samples from 624 individuals were analyzed. The results of the rapid test were compared to real-time reverse-transcription quantitative polymerase chain reaction (RT-qPCR). At the end of the test’s procedure, positive test strips were scanned in an S-Flow reader in order to roughly estimate the antigen concentration. Results: The lower limit of detection of the test was 468.75 genome copies/mL. The PROGNOSIS rapid test displayed a sensitivity of 85.5% (141/165) (95%CI: 79.1–90.5) and a specificity of 99.8% (458/459) (95%CI: 98.8–100.0%). The general inter-rater agreement was 0.89 (95%CI: 85.1–93.3). The regression analysis between the S-flow reader measurements (viral antigen) and the viral load of the positive samples demonstrated a weak correlation (R^2^ = 0.288, *p* < 0.001). Conclusion: The Rapid Test Ag 2019-nCoV demonstrated sufficient sensitivity, excellent specificity and could be available to be used with low overall cost. Thus, it could be used as point of care test, but also for mass screening for rapid detection of infected persons (e.g., for travelers).

## 1. Introduction

During the last year, the COVID-19 pandemic has influenced every country in the world. Lockdowns have been implemented with huge economic and psychological impact. Most important, as of 8 June 2021, 173,331,478 COVID-19 confirmed cases have been reported to the WHO, which resulted in 3,735,571 deaths [1]. At the same time, a total of 2,092,863,229 vaccine doses have been administered [1]; nevertheless, herd immunity seems to be distant to achieve in 2021.

More than a year since Sars-CoV-2 appeared, testing, tracking and isolation of positive cases remain basic tools to prevent the community spread of SARS-CoV-2 [2]. Real-time reverse-transcription quantitative polymerase chain reaction (RT-qPCR) is the gold standard diagnostic method for SARS-CoV-2 infection [3] due to its high sensitivity and specificity for the detection and viral load estimation of SARS-CoV-2, but it is commonly admitted that it is time-consuming, relatively expensive, and needs specialized laboratories with skilled personnel [4]. The aforementioned conditions leaded to the development and implementation of antigen-based rapid diagnostic tests (RAT) that provide results within 15–30 min without using a specialized instrument, making them ideal for point-of-care tests or mass-screening and, moreover, reduce the workload in diagnostic laboratories [5].

WHO-recommended guidelines specify a minimum of 80% sensitivity and 97% specificity for antigen-related diagnostic tests when compared with a molecular test, in order to be used for diagnosing COVID-19 patients [6]. So far, many reliable RATs have been developed, but the rapid spread of COVID-19 led to a great demand on test supplies that has created the need for local production of antigen tests.

Rapid Test Ag 2019-nCov (PROGNOSIS, BIOTECH, Larissa, Greece) is a lateral flow immunoassay designed to detect the presence or absence of nucleocapsid protein of SARS-CoV-2 in nasal or nasopharyngeal swab specimens that are directly collected. Furthermore, at the end of the test’s procedure, the test strips can be scanned in an S-Flow reader. The scanner has the ability to measure the density of the Test line, the Control line and to automatically create their ratio (T/C), giving a semi-quantitative estimation of the antigen concentration, which is a rough estimation of the viral load, which could be very helpful for the disease follow up.

The aim of the present study was:

1. To estimate the sensitivity and specificity of a local produced and recently approved RAT, Rapid Test Ag 2019-nCoV (PROGNOSIS, BIOTECH, Larissa, Greece), in a hospital setting.
2. To evaluate its accuracy for viral load determination in comparison to the reference method RT-qPCR.

## 2. Material and Methods

### 2.1. Ethics Statement

The Clinical Evaluation Study took place on the period of 19 March 2021 to 24 April 2021 at the Laboratory of Hygiene and Epidemiology of the University of Thessaly, a Biosafety Hazard 2 Laboratory, which also serves as one of the official reference laboratories for SARS-CoV-2 in Greece during the pandemic of COVID-19. The evaluation was performed in collaboration with the General Hospital of Larissa, Thessaly, Larissa. The study was approved by the scientific committee of the general hospital in accordance with national legal and ethical standards for formal approval of candidate diagnostic tests (No.: 102/19-03-2021).

### 2.2. Principal of the Assay

Rapid Test Ag 2019-nCov (PROGNOSIS, BIOTECH, Larissa, Greece) is a qualitative, lateral flow immunoassay designed to detect the presence or absence of nucleocapsid protein (N) of SARS-CoV-2 in nasal or nasopharyngeal swab specimens that are directly collected. The antigen (Nucleocapsid Protein, NP) is detectable during the acute phase of infection. Because of the conservation of N protein sequence and its strong immunogenicity, the N protein of coronavirus was chosen as a diagnostic tool [7].

**Figure 1:**
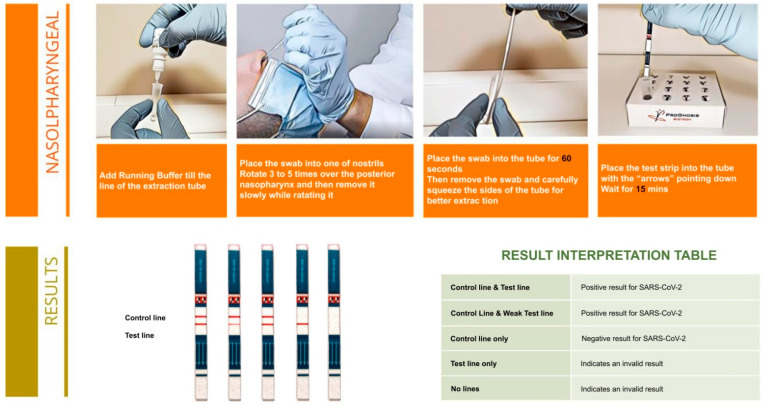
Test procedure and interpretation of the results of Rapid Test Ag 2019-nCoV (PROGNOSIS BIOTECH SA).

In this test, antibodies specific to the NP are coated on the test line region of the nitrocellulose membrane. During testing, antigens of SARS-CoV-2 in the specimen react with the antibodies that are coated onto gold nanoparticles. The mixture migrates up the membrane to react with the antibodies immobilized on the membrane and generate one colored line in the test region. The presence of this colored line indicates a positive result. To serve as a procedural control, a colored line will always appear in the control region if the test has been performed properly. The whole procedure and the interpretation of the results are demonstrated in detail in Figure 1.

### 2.3. Limit of Detection (LOD) Estimation

In order to define the LOD of the system, SARS-Related Coronavirus 2, Isolate USA-WA1/2020, Heat Inactivated was used, with a concentration of 3.75 × 10^8^ genome copies (gc)/mL as it was defined in the certificate of analysis of the strain. The reference material was deposited by the Centers for Disease Control and Prevention and was obtained through BEI Resources.

The reference material was suspended in the running buffer of the kit and dilutions of the initial solution were prepared. The dilutions and the corresponding viral loads of each dilution are shown in detail in Table 1. For each dilution, the test was performed and interpreted as described in Figure 1. In addition for each dilution, RNA extraction was performed on a KingFisher Flex System (ThermoFisher Scientific, Waltham, MA, USA) using the MagMAX™ Viral/Pathogen Nucleic Acid Isolation Kit (Applied Biosystems™, Waltham, MA USA). Finally, for the RT-PCR the TaqPath™ COVID-19 CE-IVD RT-PCR Kit (Applied Biosystems™, Waltham, MA, USA) (targets three different SARS-CoV-2 specific genomic regions; Gene ORF1ab, N Protein and S Protein) was used following the manufacturer’s instructions, on a validated QuantStudio™ 5 Real-Time PCR System (ThermoFisher Scientific, Waltham, MA, USA).

The LOD of the test was defined as the last dilution with visible colored test line. Both the rapid antigen test and the RT-PCR reaction were repeated six times for the aforementioned dilution in order to ensure the repeatability of the result.

### 2.4. Determination of Clinical Sensitivity-Specificity

Two nasopharyngeal samples from 624 individuals (all aged >18 years) were obtained simultaneously; the first from the one nostril according to the WHO guidelines for molecular analysis and the second from the other nostril according to the manufacturer’s specifications for antigen testing. In total, 276 samples were collected at the emergency ward of the General Hospital of Larissa from patients, as part of the routine diagnostic procedure. Of them, 234 were early-symptomatic patients (fever and/or cough and/or headache within 5–7 days), while 42 were asymptomatic reporting close contact to confirmed COVID-19 cases. Additionally, 348 samples from asymptomatic individuals were collected during mass screening performed by the Laboratory of Hygiene and Epidemiology. Molecular analysis was performed as described previously. Detection of SARS-CoV-2 antigens was performed on the Lateral Flow Assay using [V1301-V1310-V1330] Rapid Test Ag 2019-nCoV strictly under ProGnosis Biotech SA recommendations.

### 2.5. Reader Description and Standard Curve

**Figure 2:**
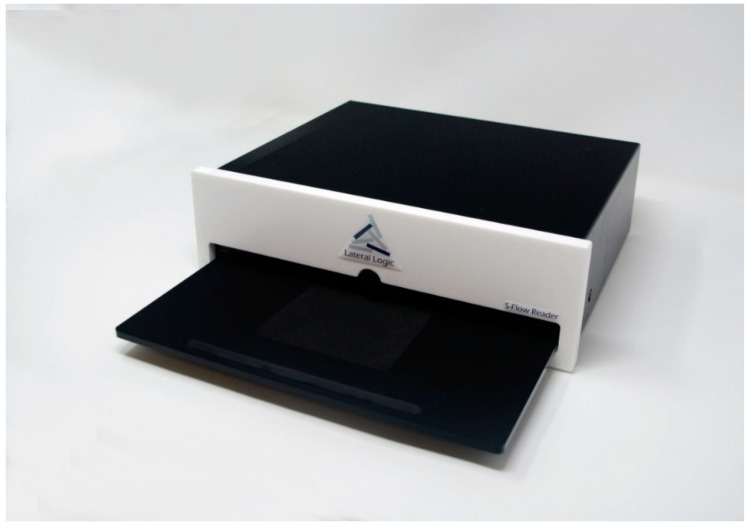
The S-Flow reader (PROGNOSIS, BIOTECH, Larissa, Greece) that was used for the measurements of the control and test lines densities.

**Figure 3:**
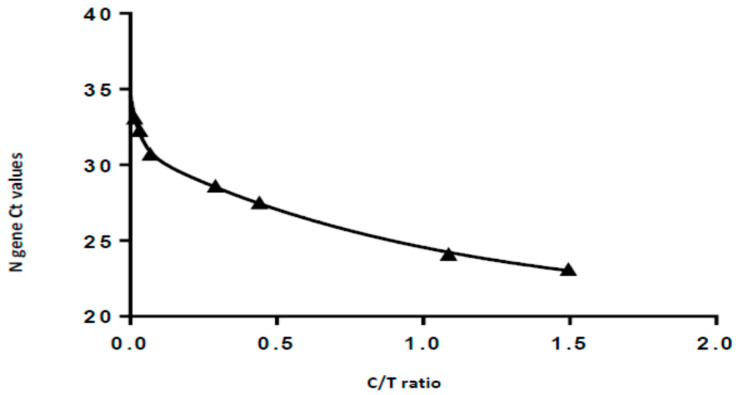
The standard curve created using N gene Ct values and the C/T ratio measurements of the S-flow reader.

In order to achieve an indirect semi-quantification of the viral load of the samples, an S-Flow reader (PROGNOSIS, BIOTECH, Larissa, Greece) was used (Figure 2). The scanner of the S-flow reader has the ability to measure the density of the Test line (T), the Control line (C) and to automatically create their ratio (T/C). Initially, a standard curve was created with the same dilutions of the reference strain that were used for the LOD determination. For each dilution, the RAT was scanned at the flow reader, and values were used in order to create the standard curve. The initial concentration of the reference strain that was used for the standard curve construction corresponded to an N gene Ct value of 22.6. The viral loads along with the Ct values of the dilutions of the reference strain, as well as the values from the S-flow reader, are demonstrated in Table 1. The standard curve that was created using the aforementioned values is shown in Figure 3.

All positive test strips from the patients of the General Hospital of Larissa were scanned in the S-Flow reader and their results were recorded. Confirmation of the dependence of the Ag test results with the viral load (for samples with N gene Ct > 22.6; 15 samples in total) was confirmed through a regression analysis between the S-Flow reader values of the strip and the corresponding Ct values of the samples.

### 2.6. Statistical Analysis

Sensitivity (Se) and specificity (Sp) were estimated with a 95% confidence interval (CI) based on binomial distribution [8]. Cohen’s kappa was calculated to evaluate the agreement between the RTD and RT-PCR [9]. Linear regression was conducted to estimate the association between the value of the RT-PCR and RTD. Statistical analysis was performed using Excel, (Microsoft, Redmond, Washington, USA) and IBM SPSS (version 25) (Microsoft, Redmond, Washington, DC, USA).

## 3. Results

### 3.1. Determination of the Lower Limit of Detection (LOD)

The RAT gave a weak colored test line for the dilution with a Ct value of 32.5 and viral load of 468.8 gc/mL. All six replicates with the aforementioned concentration that were tested with the test device were positive and confirmed the repeatability of test for the LOD.

### 3.2. Clinical Evaluation

The molecular analysis showed 165 samples were Sars-CoV-2 positive, while 459 were Sars-CoV-2 negative. In total, 141 out of 165 RT-PCR confirmed positive samples were positive with the RAT. The method displayed a sensitivity of 85.5% (141/165) (95%CI: 79.1–90.5).

Concerning the specificity of the test, 458 out of 459 RT-PCR confirmed negative samples were also negative with the RAT, which corresponds to 99.8% specificity (458/459) (95%CI: 98.8%–100.0%).

For samples with Ct values <30, the test displayed a higher sensitivity of 90.4% (141/156) (95%CI: 84.6–94.5). For the aforementioned Ct values, the positive predictive value (PPV) of the test was estimated at 80.7% for 1% prevalence and at 95.6% for 5% prevalence of COVID-19. Similarly, the negative predictive value of the rapid test was at 99.9% and 99.5%, respectively.

The general inter-rater agreement was 0.89 (95%CI: 85.1–93.3), while for the samples with Ct <30, it was 0.93 (95%CI: 89.5–96.3). The number of samples, the calculations with nominator/denominator values and the results of sensitivity and specificity of each Ct group are shown in detail in Table 2.

### 3.3. Semi-Quantification Using the Standard Curve

**Figure 4:**
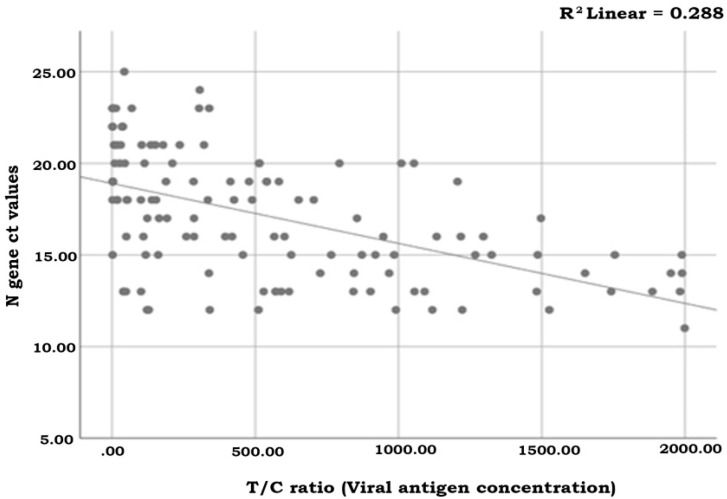
The regression analysis between the T/C ratio (viral antigen concentration) as measured by the S-flow reader and the viral load of the positive samples. A weak correlation is demonstrated since the R^2^ is equivalent to 0.288 (*p* < 0.001).

The regression analysis between the T/C ratio (viral antigen) as measured by the S-flow reader and the viral load of the positive samples, 15 positive samples with N gene Ct > 22.6 demonstrated a weak correlation since the R^2^ was equivalent to 0.288 (*p* < 0.001) (Figure 4).

## 4. Discussion

In the ongoing pandemic of COVID-19, diagnostic testing for SARS-CoV-2 is crucial to limit the spread of the virus and manage infected patients. Limitations of the gold standard nucleic acid amplification tests (NAATs) [10] promoted the development of quicker and accurate rapid diagnostic tests based on SARS-CoV-2 protein detection. Even though several tests are now available on the market, many small and low-income countries are often facing problems in obtaining the necessary tests [11]. Furthermore, the majority of commercial kits are reported to have high specificity, but a wide range of sensitivity, often lower than declared by the manufacturers, has also been reported [12,13,14,15,16,17].

In the present study, the Rapid Test Ag 2019-nCoV (ProGnosis Biotech SA) was evaluated. The evaluation was conducted in a clinical setting, the emergency ward of General Hospital of Larissa, using a large number of samples (624 in total). The LOD, sensitivity and specificity were defined. The test was able to detect low viral loads (468.75 gc/mL) and demonstrated excellent reproducibility. The ProGnosis rapid test showed a sensitivity of 85.5% (141/165) and specificity of 99.8% (458/459) when compared with RT-PCR. Previous evaluations of the most common commercial rapid antigen tests reported similar or inferior results [18]. In the study of Fourati et al., SARS-CoV-2 COVID-19 Respi-Strip (Coris BioConcept, Gembloux, Belgique) and NG Test SARS-CoV-2 Ag (NG Biotech, Guipry, France) showed poor sensitivity (42.6% and 38.9%, respectively, for Ct values < 33); Standard Q COVID-19 Ag (SD BIOSENSOR, Inc., Coree), Biosynex COVID-19 Ag BSS (Biosynex, Strasbourg, France) and COVID-VIRO Antigen Rapid Test (AAZ, Boulogne-Billancourt, France) tests showed a relatively satisfactory total sensitivity (55–62%) that reached 87–96% for Ct values <25 [18]. Concerning specificity, CORIS, ABBOTT, NG BIOTECH and AAZ reached 100%, and BIOSENSOR and BIOSYNEX reached 93.2% and 98.5%, respectively [18].

On the other hand, when the Rapid Test Ag 2019-nCoV (ProGnosis Biotech SA) was used for antigen semi-quantification with an S-flow reader, the correlation demonstrated by linear regression analysis was weak (R^2^ = 0.288). Since in the recent literature there are studies [19] supporting the potential of rapid semi-quantification of the viral load using RAT, the improvement of the test towards that direction remains a future challenge.

The main limitation we encountered was that when the study took place, 19 March 2021 to 24 April 2021, the vast majority of the variants circulating in Larissa were Alpha (B.1.1.7, VOC-20DEC-01) and to a lesser degree B.1.177. The RAT was able to identify both variants, but the constant evaluation of rapid antigen tests, in general, is required as new variants emerge.

In conclusion, Rapid Test Ag 2019-nCoV (ProGnosis Biotech SA) showed an overall sensitivity of 85.5% and an inter-rater agreement (weighted Kappa) when compared with RT-PCR of 0.89, indicating that it could accurately identify SARS-CoV-2 antigens in people with suspected COVID-19. The fact that it is manufactured in Greece makes the rapid test easily accessible for the Greek healthcare settings and helps address deficiencies that may arise due to increased demand for diagnostic tests, especially in high prevalence areas.

## Figures and Tables

**Table 1:** Viral load and N gene Ct values of the reference strain dilutions used for the evaluation of the Rapid Test Ag 2019-nCoV (ProGnosis Biotech SA). Corresponding densities of the test line (T), the control line (C) and their ratio (T/C) as estimated by the S-flow reader used for the creation of the standard curve.

	Viral Load (gc/mL)	N Gene Ct Value	Test Line Density (T)	Control Line Density (C)	Ratio T/C
1	187 500	22.6	387.817	266.465	1.452
2	18 750	27.5	111.206	252.339	0.441
3	1 875	30.7	16.153	237.341	0.068
4	937.5	31.6	10.191	226.687	0.045
5	468.8	32.5	7.152	217.968	0.033
6	0 (negative control)	undetermined	0.255	203.568	0.001

**Table 2:** Specificity, sensitivity, PPN, NPV and inter-rater agreement of the test according to Ct values and different prevalence rates of the disease.

	Sensitivity (%)				Specificity (%)				Inter-Rater Agreement (Kappa)			PPV (%)			NPV (%)		
	Positive Samples (*n*)		95% CI		Negative Samples (n)		95% CI			95% CI		Prevalence			Prevalence		
												1‰	1%	5%	1‰	1%	5%
Total	141/165	85.5	79.1	90.5	458/459	99.8	98.8	100.0	0.89	85.1	93.3	28.2	79.9	95.4	99.99	99.9	99.2
Ct < 30	141/156	90.4	84.6	94.5					0.93	89.5	96.3	28.6	80.7	95.6	99.99	99.9	99.5

Sensitivity, which is the probability that a test result will be positive when the disease is present (true positive rate) was calculated for each group according to the following type: a/(a + b). Specificity, which is the probability that a test result will be negative when the disease is not present (true negative rate) was calculated according to the following type: d/(c + d). Positive predictive value, which is the probability that the disease is present when the test is positive was calculated for each group according to the following type: PPV = Se × Prevalence/Se × Prevalence + (1-Sp) × (1-Prevalence). Negative predictive value, which is the probability that the disease is not present when the test is negative was calculated for each group according to the following type: NPV = Sp × (1-Prevalence)/(1-Se) × Prevalence + Sp × (1-Prevalence). Inter-rater agreement, which is the evaluation of the agreement between the two classifications. The weighted Kappa according to Cohen, 1968 was calculated.

## Data Availability

Data is available upon request from the corresponding author.
